# pFAK-Y397 overexpression as both a prognostic and a predictive biomarker for patients with metastatic osteosarcoma

**DOI:** 10.1371/journal.pone.0182989

**Published:** 2017-08-28

**Authors:** Kamolrat Thanapprapasr, Adisak Nartthanarung, Duangmani Thanapprapasr, Artit Jinawath

**Affiliations:** 1 Biomedical Engineering Research Unit, National Metal and Materials Technology Center, National Science and Technology Development Agency, Pathumthani, Thailand; 2 Musculoskeletal Oncology Unit, Department of Orthopaedics, Faculty of Medicine Ramathibodi Hospital, Mahidol University, Bangkok, Thailand; 3 Surgical Oncology Unit, Wattanosoth Cancer Hospital, Bangkok Hospital Medical Center, Bangkok, Thailand; 4 Department of Pathology, Faculty of Medicine Ramathibodi Hospital, Mahidol University, Bangkok, Thailand; University of South Alabama Mitchell Cancer Institute, UNITED STATES

## Abstract

Focal adhesion kinase (FAK) is important for tumor cell survival and metastasis in various cancers. However, its expression and prognostic value in patients with metastatic osteosarcoma remain unknown. We investigated the expression of FAK and its phosphorylated form (pFAK-Y397) in osteosarcoma tissues from 53 patients by immunohistochemistry and evaluated their correlations with clinicopathologic characteristics and outcomes. The prognostic values were assessed using Kaplan-Meier survival and Cox regression analyses. Total FAK and pFAK-Y397 were overexpressed in 48 (90.6%) and 33 (62.3%) cases, respectively. pFAK-Y397 overexpression was correlated with poor histologic response after neoadjuvant chemotherapy in patients with osteosarcoma regardless of the presence of metastasis or not. Kaplan-Meier curve showed that patients with metastatic osteosarcoma with pFAK-Y397 overexpression had significantly worse overall survival (OS) than those with non-overexpression (*P* = 0.044). Multivariate Cox regression analysis confirmed pFAK-Y397 overexpression as an independent prognostic predictor for OS and post metastases OS (PMOS) (*P* = 0.017, *P* = 0.006, respectively). Age at diagnosis was also an independent indicator for PMOS (*P* = 0.003). However, total FAK expression was not correlated with any clinicopathologic characteristics or OS in patients with metastatic osteosarcoma. In conclusion, our findings identified FAK as a common aberrant protein overexpression in various subtypes of osteosarcoma. pFAK-Y397 overexpression can be used as a prognostic biomarker predicting poor OS for patients with metastatic osteosarcoma, and the expression of pFAK-Y397 differentiated good and poor responders to neoadjuvant chemotherapy.

## Introduction

Osteosarcoma is the most common primary malignant bone tumor affecting mainly children and young adults [1]. However, its incidence has also been increasing in the middle-aged people over 40 years in some countries [2]. Development of fatal metastasis such as pulmonary metastasis remains the most significant poor prognosis of this disease. With the introduction of adjuvant or neoadjuvant chemotherapy to surgery, the survival rates of metastatic-free patients have rapidly improved up to nearly 80%, but no major survival improvement has been reported since the 1970s [1,3,4]. On the other hand, the prognosis of patients with metastatic disease at diagnosis remains extremely poor even though potent drugs such as ifosfamide and methotrexate are employed in the treatment [5]. Regrettably, patients with metastatic disease at diagnosis were usually treated with the same conventional chemotherapy protocols as patients with non-metastatic disease, which resulted in very poor outcome [5,6]. Therefore, it is imperative to explore new prognostic predictors and novel therapeutic approaches for patients with osteosarcoma, particularly for patients with metastatic disease at diagnosis.

Focal adhesion kinase (FAK), a non-receptor cytoplasmic protein tyrosine kinase, has been reported to be overexpressed in many cancer types and implicated in many cell signaling pathways leading to cell proliferation, invasion, survival and metastasis [7–11]. Moreover, nuclear-localized form of FAK may process unique functions, and its expression was associated with patient’s survival in some tumors [12,13]. The major site of autophosphorylation in the FAK catalytic domain at tyrosine 397 is very important for FAK function leading to many downstream signaling cascades of cell proliferation, migration, and angiogenesis [14,15]. Recently, in order to inhibit FAK activity, a number of small molecule ATP-competitive kinase inhibitors have been developed, as well as more specific inhibitors, that target particular kinase domain sites of FAK by blocking access to the FAK at tyrosine 397. These inhibitors prevented cell adhesion, caused apoptosis and decreased tumor growth in preclinical models [10,14,16]. In the future, FAK-targeted therapy might become a novel approach for cancer treatment as well as prevention of tumor metastasis in cancer patients.

Many previous studies reported that expression of total (phosphorylated and nonphosphorylated) FAK and/or the phosphorylated form of FAK at tyrosine 397 (pFAK-Y397) can be prognostic predictors in various types of malignant tumors including colorectal cancer [13], non-small-cell lung carcinoma [17], breast carcinoma [18], hepatocellular carcinoma [19,20], cervical cancer [21], glioma [22], epithelial ovarian carcinoma [23], endometrial carcinoma [24], and gastric cancer [25]. To our knowledge, only a few studies of FAK expression in patients with osteosarcoma have previously been published, and they excluded patients with metastatic osteosarcoma at diagnosis from their studies [26,27]. None of them has specifically addressed the scopes of prognostic value of FAK expression and its correlation with response to chemotherapy of patients with metastatic osteosarcoma at diagnosis or during treatment/follow-up. Prognostic value of pFAK-Y397 and its correlated clinicopathologic characteristics could be worthy for development of more effective treatment regimens and personalized treatments for patients with metastatic osteosarcoma. The aims of this study were to investigate the expression of total FAK and pFAK-Y397 by immunohistochemistry in pretreatment osteosarcoma tumors of patients with various histologic subtypes, and to investigate correlations of FAK expression with response to chemotherapy, other clinicopathologic characteristics, overall survival (OS), and survival after metastases of all osteosarcoma patients with or without metastasis. We hypothesized that overexpression of pFAK-Y397 alone would be a significant biomarker in prognostic assessment of patients with metastatic osteosarcoma, and a potential predictor of response to neoadjuvant chemotherapy.

## Materials and methods

### Patients and tissue samples

This study was a retrospective analysis approved by the Committee on Human Rights Related to Research Involving Human Subjects, Faculty of Medicine Ramathibodi Hospital, Mahidol University. The committee waived the need for consent. A total of 71 patients with osteosarcoma diagnosed at Ramathibodi Hospital, Bangkok, Thailand, during April 2005 to March 2014 were enrolled. Patients with incomplete medical records (3 patients), unfound tissues (10 patients), and low grade central and extraskeletal osteosarcoma (5 patients) were excluded. Finalized data of 53 patients with high-grade primary osteosarcoma were collected. Patient clinicopathologic characteristics including age at diagnosis, gender, primary tumor volume, site of tumor, histologic subtype, presence of metastasis at diagnosis, site of metastases, time to metastases, histologic response to neoadjuvant chemotherapy treatment (percentage of tumor necrosis), and neoadjuvant and adjuvant chemotherapy treatment were recorded and analyzed. Tumor volume could be retrospectively evaluated only in 45 patients by preoperative magnetic resonance image (MRI), and calculated by the ellipsoid formula: (4π;/3)abc = (π/6) × tumor width × tumor length × tumor vertical height [28]. Total FAK and pFAK-Y397 expression in the osteosarcoma tissues were characterized. Correlations between total FAK, pFAK-Y397 expression and the clinicopathologic characteristics and survival were evaluated. FAK overexpression and OS were the main focus of this study. The duration of OS was determined from the date of primary tumor diagnosis to either the date of death or the surviving cutoff date for analysis on October 11, 2016. Post metastases overall survival (PMOS) or survival after metastases was defined as the duration from the time of first detection of metastases until the date of death or the surviving cutoff date.

### Immunohistochemistry

Fifty three formalin-fixed paraffin embedded (FFPE) tissues of 53 patients with osteosarcoma before starting chemotherapy were sectioned at 4μm and stained with the mouse anti-human FAK and pFAK-Y397 antibodies (BD Transduction Laboratories, US) at 1:25 dilution using Bond Polymer Refine detection kit on Bond-Max automated immunostainer (Leica Microsystems, Milton Keynes, UK) according to the manufacturer’s protocol.

### Evaluation of immunohistochemical stainings

Immunoreactivity was evaluated semi-quantitatively by assessing the percentage of positive staining and staining intensity in tumor cells. The percentage of positive cells was determined as follows: 0, no staining; 1, < 25%; 2, 25–49%; 3, 50–74%; and 4, 75–100%. The staining intensity was rated as follows: 0, none; 1, weak; 2, moderate; 3, strong. The staining was marked as follows: N, nuclear and cytoplasmic; C, cytoplasmic. The scores from staining percentage and intensity were added to yield the overall scores ranging from 0 to 7. To define total FAK or pFAK-Y397 expression, patients were classified into four groups. The overall scores of 0, 1–2, 3–4, and 5–7 were classified as negative expression, weak expression, moderate expression and overexpression, respectively.

### Statistical analysis

Correlations between total FAK or pFAK-Y397 expression and clinicopathologic characteristics of patients with osteosarcoma were analyzed using the Chi-square or the Fisher’s exact tests where appropriate. Survival curves between divided groups of total FAK, pFAK-Y397 expression, their co-expression, and other clinicopathologic characteristics were created using the Kaplan-Meier method. The log-rank (Mantel-Cox) test was performed to compare differences in OS and PMOS curves of patients. The two-sided *P* value of less than 0.05 was considered statistically significant. Independent prognostic factors were identified by the Cox proportional hazards regression model analyses in a stepwise manner. All variables considered significant at the prognostic value less than 0.05 level which related to patient survival in the univariate Cox regression analyses were included in the multivariate models. All statistical analyses were performed using the IBM SPSS Statistics version 22.0 software (IBM Corporation, USA).

## Results

### Clinicopathologic characteristics of patient tissue samples

All 53 tumor tissue samples were collected from patients diagnosed with osteosarcoma before any treatment. Of the 53 patients, 36 were males (67.9%) and 17 were females (32.1%) with the mean age at diagnosis of 18 years (median, 12 years; range, 5–67 years). By the Enneking Surgical Staging System, 19 patients (35.9%) had a stage III tumor, 34 (64.1%) had a stage IIB tumor and no patient had a stage IIA tumor in this study. Primary tumor localizations were extremities in 52 patients (98.1%), and axial in one patient (1.9%; in the pelvis). The most frequent site was distal femur (27 patients, 50.9%). The primary tumor volume ranged from 28 to 2,659 mL with a mean of 520 mL. Nineteen of 45 patients (42.2%) had tumor volume larger than 500 mL. The two most frequent histologic subtypes were osteoblastic (28 patients, 52.8%) and chondroblastic (13 patients, 24.5%). Fibroblastic, telangiectatic and small cell osteosarcomas were also included in our study (7, 4 and 1 patients; 13.2, 7.5 and 1.9%, respectively).

Metastasis presented at the time of diagnosis in 19 patients (35.9%), and presented during treatment/follow-up in 14 patients (26.4%), whereas 12 patients (22.6%) did not develop metastasis until the cutoff date and the remaining 8 patients were lost to follow-up. Metastasis at diagnosis was detected in a single site: lung only (13 patients, 39.4%) and multiple sites: multiple organs (1 patient, 3.0%, lung, liver and bone; 1 patient, 3.0%, lung and liver; 2 patients, 6.1%, lung and bone; 1 patient, 3.0%, lung and brain), and multiple bones (1 patient, 3.0%, spine and sacrum). Metastasis during treatment/follow-up was detected in lung only (12 patients, 36.4%) and multiple sites (1 patient, 3.0%, multiple bones; 1 patient, 3.0%, lung and liver). First metastases were detected: at diagnosis (19 patients, 57.6%), ≤ 12 months (6 patients, 18.2%) and > 12 months (8 patients, 24.2%). However, metastatic tissue was excluded from the study.

All patients received appropriate surgical and chemotherapeutic treatment. The main primary tumor surgical procedure that patients underwent was amputation (30 patients, 56.6%). The procedures of the remaining patients were limb-sparing (8 patients, 15.1%), disarticulation (5 patients, 9.4%), rotationplasty (1 patient, 1.9%) and 9 patients (17.0%) were treated with no surgery. Chemotherapy was given to the patients according to the standard chemotherapeutic regimens for osteosarcoma at the time of enrollment. Chemotherapeutic protocols consisted of combinations of drugs such as methotrexate, doxorubicin, cisplatin, etoposide, cyclophosphamide, ifosfamide and carboplatin. Thirty four patients (64.2%) received both neoadjuvant and adjuvant chemotherapy, others received neoadjuvant (9 patients, 17.0%), adjuvant (5 patients, 9.4%), and no chemotherapy (5 patients, 9.4%) in this study. The good and poor histologic response to neoadjuvant chemotherapy were defined as having ≥ 90% or < 90% tumor necrosis rate, respectively [29]. Of the 35 patients with adequate tumor necrosis data, 13 were good responders to neoadjuvant chemotherapy (37.1%) and 22 were poor responders (62.9%). Sixteen of 53 patients were still alive on the surviving cutoff date (12 patients, 22.6%, without metastatic disease until the cutoff date; 3 patients, 5.7%, with metastases during treatment/follow-up; 1 patient, 1.9%, with metastases at diagnosis).

### Expression of total FAK and pFAK-Y397 in osteosarcoma tissues

**Fig 1:**
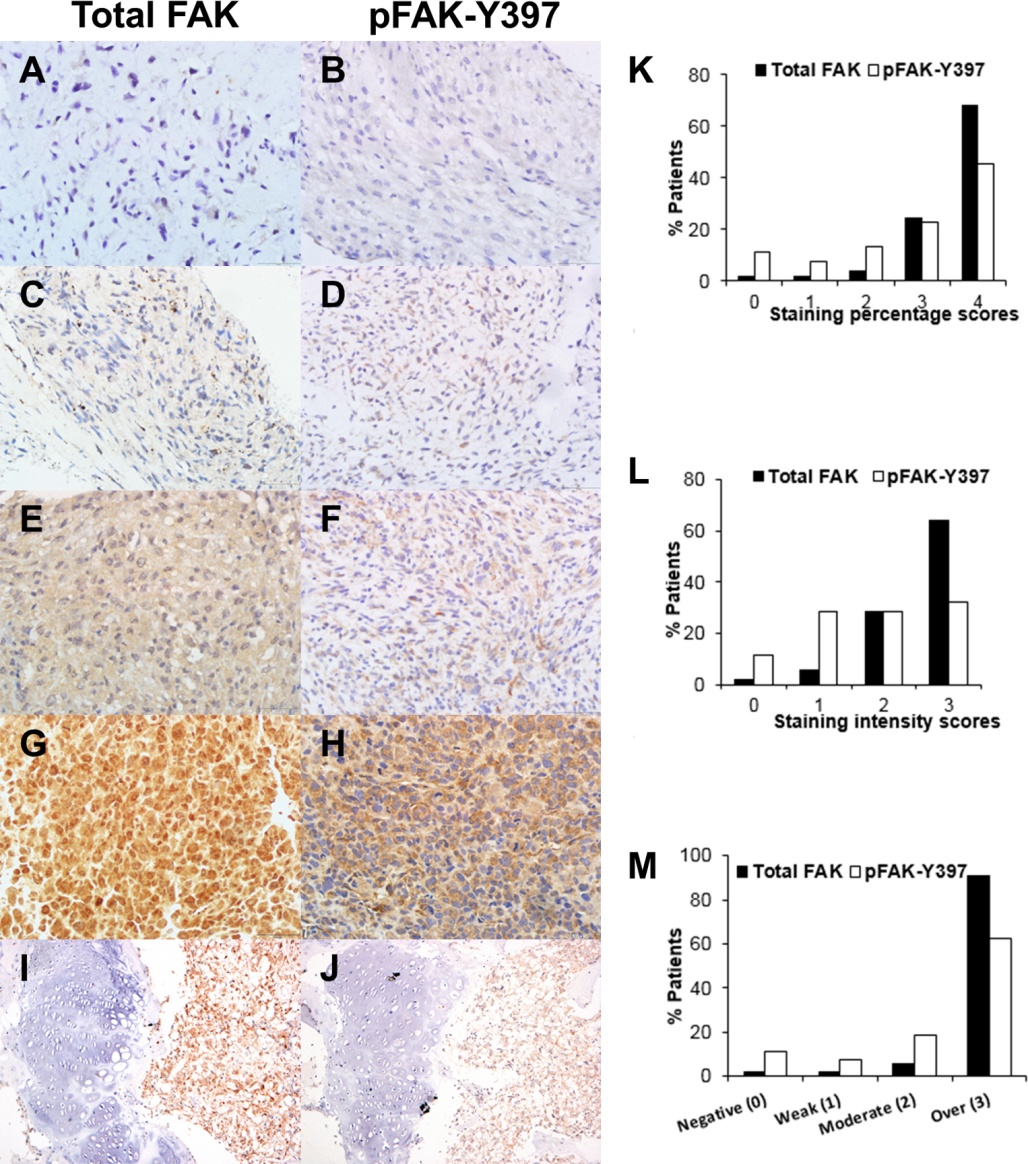
Immunohistochemical analysis of total FAK and pFAK-Y397 expression in osteosarcoma tissues. (A, B) Negative expression, (C, D) weak expression, (E, F) moderate expression, (G, H) strong expression. (I, J) Strongly diffused total FAK (I-right) or pFAK-Y397 (J-right) expression in osteosarcoma, in contrast to diminished total FAK (I-left) or pFAK-Y397 (J-left) expression in adjacent epiphyseal growth plates. Original magnifications, x400 (A-H), x100 (I, J). Analysis of immunohistochemistry of total FAK and pFAK-Y397. (K) Percentage of positive tumor cells classified into 4 categories: 0, no staining; 1, < 25%; 2, 25–49%; 3, 50–74%; 4, 75–100%. (L) Staining intensity of positive tumor cells: 0, none; 1, weak; 2, moderate; 3, strong. (M) Overall score for total FAK or pFAK-Y397 expression in patients with osteosarcoma. The scores from staining percentage and intensity were added to yield the overall scores of 0, negative; 1–2, weak; 3–4, moderate; 5–7, overexpression.

To determine the expression and localization of FAK, immunohistochemical analyses of total FAK and pFAK-Y397 in osteosarcoma tissues obtained from 53 patients were performed. Representative images of osteosarcoma tissue samples with negative, weak, moderate, and strong staining intensities of total FAK and pFAK-Y397 are shown in Fig 1A*-*1H.

Fifty two out of 53 (98.1%) of osteosarcoma tissue samples had detectable total FAK expression and 47 out of 53 (88.7%) of the samples had detectable pFAK-Y397 expression with different percentages and intensities of positive cells Fig 1K and 1L. The scores of both percentage and intensity of total FAK and pFAK were combined to create the final overall FAK expression score. The total FAK was overexpressed in 48 (90.6%) tissue samples while the pFAK-Y397 was overexpressed in 33 (62.3%) tissue samples (Fig 1M). Negative, weak, or moderate expression levels of the total FAK were observed in 1 (1.9%), 1 (1.9%), and 3 (5.7%) tissue samples, respectively. In contrast, negative, weak or moderate expression levels of pFAK-Y397 were detected in 6 (11.3%), 4 (7.5%), and 10 (18.9%) tissue samples, respectively. The staining patterns for total FAK were both nuclear and cytoplasmic but for pFAK-Y397 the staining was predominantly cytoplasmic. Fig 1G and 1H) clearly illustrates total FAK expression in the nuclei and cytoplasm, and pFAK-Y397 expression in cytoplasm. In almost all cases, the staining intensities of pFAK-Y397 in tumors were lower than that of total FAK. Our results showed that both total FAK and pFAK-Y397 were significantly overexpressed in the majority of human osteosarcomas. In addition, the staining patterns and intensities for total FAK or pFAK-Y397 as described above were observed in all histologic subtypes of tissue samples in this study.

Expression of pFAK-Y397 in the nuclei was focally observed in very few cases of osteosarcoma in this study. In matched normal tissues, total FAK and pFAK-Y397 staining were absent or focally weak positive in blood vessels, reactive bones and epiphyseal growth plates as shown in Fig 1I and 1J.

### Total FAK and pFAK-Y397 overexpression in various subtypes of osteosarcoma

In our study, overexpression of FAK was detected in conventional (osteoblastic, chondroblastic, and fibroblastic subtypes) and rare subtypes of osteosarcoma including telangiectatic and small cell types. The total FAK was overexpressed in 26 out of 28 osteoblastic, 13 out of 13 chondroblastic, 5 out of 7 fibroblastic, 3 out of 4 telangiectatic and 1 out of 1 small cell osteosarcoma. Also, the pFAK-Y397 was overexpressed in 18 out of 28 osteoblastic, 8 out of 13 chondroblastic, 3 out of 7 fibroblastic, 3 out of 4 telangiectatic and 1 out of 1 small cell osteosarcomas.

### Relationships between total FAK or pFAK-Y397 expression and clinicopathologic characteristics

The relationships between total FAK or pFAK-Y397 expression and clinicopathologic characteristics of all the 53 patients with osteosarcoma and 33 patients with metastatic osteosarcoma at diagnosis or developed metastases during treatment/follow-up, were statistically analyzed as summarized in Table 1. In the group of all patients, these analyses revealed significant correlations between total FAK or pFAK-Y397 overexpression and histologic response to neoadjuvant chemotherapy (good vs poor, *P* = 0.014 and 0.0004, respectively) of 35 patients who received neoadjuvant chemotherapy. However, in the group of patients with metastatic osteosarcoma, we found that pFAK-Y397 overexpression was significantly correlated with histologic response to neoadjuvant chemotherapy (*P* = 0.004), while total FAK expression was not (*P* = 0.238). Moreover, significant correlations between total FAK or pFAK-Y397 overexpression and histologic response to neoadjuvant chemotherapy were also found in patients without metastatic osteosarcoma at diagnosis (*n* = 34) in this study (*P* = 0.037 and 0.009, respectively, S1 Table). However, there was no statistically significant correlation between total FAK or pFAK-Y397 expression and other characteristics (*P* > 0.05) in both groups of all patients and patients with metastatic osteosarcoma. The results suggest that pFAK-Y397 is commonly overexpressed in patients with osteosarcoma and its overexpression is correlated with poor histologic response to chemotherapy of patients with osteosarcoma regardless of the presence of metastasis or not.

**Table 1:** Relationships between clinicopathologic characteristics and total FAK, pFAK-Y397 expression in primary osteosarcoma tissues of all patients with osteosarcoma, and patients with osteosarcoma presented with metastases at diagnosis or developed metastases during treatment/follow-up. (non-overexpression = negative or weak or moderate expression)

Characteristics	All patients with osteosarcoma (*n* = 53)						Patients with metastatic osteosarcoma (*n* = 33)					
	Total FAK expression			pFAK-Y397 expression			Total FAK expression			pFAK-Y397 expression		
	Non-over (*n* = 5) *n* (%)	Over (*n* = 48) *n* (%)	*P* value^e^	Non-over (*n* = 20) *n* (%)	Over (*n* = 33) *n* (%)	*P* value^e^	Non-over (*n* = 2) *n* (%)	Over (*n* = 31) *n* (%)	*P* value^e^	Non-over (*n* = 12) *n* (%)	Over (*n* = 21) *n* (%)	*P* value^e^
Age at diagnosis^a^												
≤ 18 yrs	4 (7.5)	38 (71.7)	1.000	16 (30.2)	26 (49.1)	1.000	1 (3.0)	26 (78.8)	0.335	9 (27.3)	18 (54.5)	0.643
> 18 yrs	1 (1.9)	10 (18.9)		4 (7.5)	7 (13.2)		1 (3.0)	5 (15.2)		3 (9.1)	3 (9.1)	
Gender												
Male	4 (7.5)	32 (60.4)	1.000	14 (26.4)	22 (41.5)	0.801	1 (3.0)	26 (78.8)	0.335	10 (30.3)	17 (51.5)	1.000
Female	1 (1.9)	16 (30.2)		6 (11.3)	11 (20.8)		1 (3.0)	5 (15.2)		2 (6.1)	4 (12.1)	
Site of tumor												
Femur	4 (7.5)	27 (50.9)	0.389	11 (20.8)	20 (37.7)	0.688	2 (6.1)	14 (42.4)	0.227	6 (18.2)	10 (30.3)	0.895
Tibia or fibula or others	1 (1.9)	21 (39.6)		9 (17.0)	13 (24.5)		0 (0.0)	17 (51.5)		6 (18.2)	11 (33.3)	
Histologic subtype												
Non osteoblastic	3 (5.7)	22 (41.5)	0.658	10 (18.9)	15 (28.3)	0.748	1 (3.0)	15 (45.5)	1.000	6 (18.2)	10 (30.3)	0.895
Osteoblastic	2 (3.8)	26 (49.1)		10 (18.9)	18 (34.0)		1 (3.0)	16 (48.5)		6 (18.2)	11 (33.3)	
Primary tumor volume^b^												
< 500 mL	2 (4.4)	24 (53.3)	1.000	11 (24.4)	15 (33.3)	0.268	0 (0.0)	12 (46.2)	1.000	5 (19.2)	7 (26.9)	0.401
≥ 500 mL	2 (4.4)	17 (37.8)		5 (11.1)	14 (31.1)		1 (3.8)	13 (50.0)		3 (11.5)	11 (42.3)	
Presence of metastasis at diagnosis												
No	4 (7.5)	30 (56.6)	0.643	13 (24.5)	21 (39.6)	0.920	1 (3.0)	13 (39.4)	1.000	5 (16.1)	9 (27.3)	0.947
Yes	1 (1.9)	18 (34.0)		7 (13.2)	12 (22.6)		1 (3.0)	18 (54.5)		7 (22.6)	12 (36.4)	
Time to metastases^c^												
> 12 months	0 (0.0)	8 (24.2)	0.388	3 (9.1)	5 (15.2)	1.000	0 (0.0)	8 (24.2)	0.388	3 (9.1)	5 (15.2)	1.000
≤ 12 months	1 (3.0)	5 (15.2)		2 (6.1)	4 (12.1)		1 (3.0)	5 (15.2)		2 (6.1)	4 (12.1)	
At diagnosis	1 (3.0)	18 (54.5)		7 (21.2)	12 (36.4)		1 (3.0)	18 (54.5)		7 (21.2)	12 (36.4)	
Site of metastases^c^												
Lung only	1 (3.0)	24 (72.7)	0.432	10 (30.3)	15 (45.5)	0.678	1 (3.0)	24 (72.7)	0.432	10 (30.3)	15 (45.5)	0.678
Bone or multiple organs	1 (3.0)	7 (21.2)		2 (6.1)	6 (18.2)		1 (3.0)	7 (21.2)		2 (6.1)	6 (18.2)	
Histologic response^d^												
Good	4 (11.4)	9 (25.7)	0.014*	9 (25.7)	4 (11.4)	0.0004*	1 (4.8)	4 (19.0)	0.238	4 (19.0)	1 (4.8)	0.004*
Poor	0 (0.0)	22 (62.9)		2 (5.7)	20 (57.1)		0 (0.0)	16 (76.2)		1 (4.8)	15 (71.4)	

^a^ Mean value of age at diagnosis: patients with metastatic osteosarcoma, ≤ 21 yrs vs > 21 yrs.

^b^ All patients with osteosarcoma, evaluated only in 45 patients (8 patients, no preoperative MRI); patients with metastatic osteosarcoma, evaluated only in 26 patients (7 patients, no preoperative MRI).

^c^ All patients with osteosarcoma, evaluated only in 33 patients (12 patients, no metastasis until the cutoff date; 8 patients, loss to follow-up).

^d^ All patients with osteosarcoma, evaluated only in 35 patients who received neoadjuvant chemotherapy (5 patients, no neoadjuvant chemotherapy; 5 patients, no chemotherapy; 8 patients, no data); patients with metastatic osteosarcoma, evaluated only in 21 patients who received neoadjuvant chemotherapy (5 patients, no neoadjuvant chemotherapy; 5 patients, no chemotherapy; 2 patients, no data).

^e^ *P* value determined using the Pearson Chi-square test or Fisher’s exact test.

* Statistically significant.

### Association of FAK expression and other clinicopathological characteristics with the survival

To identify patient characteristics that might be statistically correlated with survival of patients with osteosarcoma, we examined clinicopathologic characteristics including total FAK, pFAK-Y397 expression, total FAK/pFAK-Y397 co-expression and other characteristics using Kaplan-Meier survival analysis and the log-rank test. The results of Kaplan-Meier survival analyses of patients with osteosarcoma are summarized in Table 2.

**Table 2:** Kaplan-Meier survival analyses for overall survival of all patients with osteosarcoma and for overall survival and post metastases overall survival of patients with osteosarcoma presented with metastases at diagnosis or developed metastases during treatment/follow-up.

Characteristics	All patients with osteosarcoma (*n* = 53)			Patients with metastatic osteosarcoma (*n* = 33)					
	OS			OS			PMOS		
	*n*	Mean months (95% CI)	*P* value^d^	*n*	Mean months (95% CI)	*P* value^d^	*n*	Mean months (95% CI)	*P* value^d^
Age at diagnosis^a^									
≤ 18 yrs	42	56 (40.9–70.9)	0.013*	27	34 (23.9–43.3)	0.062	27	24 (15.8–32.4)	0.011*
> 18 yrs	11	19 (8.4–28.9)	6	16 (3.1–28.4)			6	9 (3.3–15.2)	
Gender									
Male	36	51 (34.7–66.8)	0.780	27	32 (21.6–42.0)	0.485	27	23 (14.0–31.4)	0.389
Female	17	45 (24.2–66.2)		6	24 (16.1–31.8)		6	16 (8.9–23.5)	
Site of tumor									
Femur	31	58 (39.2–77.2)	0.326	16	32 (20.5–43.7)	0.661	16	26 (14.7–37.6)	0.208
Tibia or fibula or others	22	40 (24.2–55.0)		17	28 (16.5–39.6)		17	16 (9.0–23.6)	
Histologic subtype									
Non osteoblastic	25	62 (41.1–81.9)	0.102	16	36 (24.0–47.9)	0.140	16	22 (15.3–28.7)	0.451
Osteoblastic	28	37 (22.6–51.7)		17	24 (13.8–33.4)		17	19 (8.9–29.0)	
Primary tumor volume^b^									
< 500 mL	26	69 (49.2–89.3)	0.002*	12	43 (26.4–59.6)	0.074	12	28 (12.6–43.8)	0.374
≥ 500 mL	19	24 (15.2–33.0)		14	24 (14.4–32.6)		14	20 (11.9–27.1)	
Presence of metastasis at diagnosis									
No	34	65 (47.1–82.8)	0.0003*	14	41 (28.5–52.8)	0.037*	14	18 (12.3–24.4)	0.882
Yes	19	22 (12.5–30.7)		19	22 (12.5–30.7)		19	21 (12.1–30.4)	
Time to metastases									
> 12 months				8	53 (37.1–69.4)	0.028*	8	21 (11.9–29.4)	0.688
≤ 12 months				6	25 (16.6–33.1)		6	16 (7.1–25.0)	
At diagnosis				19	22 (12.5–30.7)		19	21 (12.1–30.4)	
Site of metastases									
Lung only				25	34 (24.0–44.7)	0.050	25	25 (15.6–33.6)	0.019*
Bone or multiple organs				8	18 (8.5–26.5)		8	12 (6.7–16.4)	
Histologic response^c^									
Good	13	87 (59.2–114.7)	0.024*	5	37 (17.0–57.5)	0.379	5	25 (7.5–42.3)	0.419
Poor	22	41 (27.4–53.8)		16	29 (19.6–38.8)		16	20 (14.1–25.2)	
Chemotherapy									
Both neoadjuvant & adjuvant	34	65 (48.0–81.8)	0.00003*	21	34 (25.0–43.8)	0.133	21	23 (16.6–29.0)	0.183
Either neoadjuvant or adjuvant, or no chemotherapy	19	20 (9.4–30.0)		12	22 (8.3–35.1)		12	17 (3.8–29.9)	
Total FAK expression									
Non-over	5	66 (21.1–109.9)	0.449	2	29 (17.5–40.9)	0.958	2	21 (15.2–26.6)	0.962
Over	48	45 (32.6–57.0)		31	30 (21.3–39.4)		31	21 (13.8–29.0)	
pFAK-Y397 expression									
Non-over	20	60 (37.5–82.5)	0.258	12	41 (23.2–58.1)	0.044*	12	32 (14.9–48.6)	0.029*
Over	33	40 (26.2–54.6)		21	24 (16.4–32.1)		21	15 (11.5–19.4)	
Total FAK/pFAK-Y397 co-expression									
Non-over/non-over	5	66 (21.1–109.9)	0.502	2	29 (17.5–40.9)	0.098	2	21 (15.2–26.6)	0.071
Over/non-over	15	49 (29.0–68.3)		10	43 (22.3–63.6)		10	34 (14.0–53.8)	
Over/over	33	40 (26.2–54.6)		21	24 (16.4–32.1)		21	15 (11.5–19.4)	

CI, confidence interval; OS, overall survival; PMOS, post metastases overall survival.

^a^ Mean value of age at diagnosis: patients with metastatic osteosarcoma, ≤ 21 yrs vs > 21 yrs.

^b^ All patients with osteosarcoma, evaluated only in 45 patients (8 patients, no preoperative MRI); patients with metastatic osteosarcoma, evaluated only in 26 patients (7 patients, no preoperative MRI).

^c^ All patients with osteosarcoma, evaluated only in 35 patients who received neoadjuvant chemotherapy (5 patients, no neoadjuvant chemotherapy; 5 patients, no chemotherapy; 8 patients, no data); patients with metastases, evaluated only in 21 patients who received neoadjuvant chemotherapy (5 patients, no neoadjuvant chemotherapy; 5 patients, no chemotherapy; 2 patients, no data).

^d^ Log rank (Mantel-Cox) test.

* Statistically significant.

In the group of all patients with osteosarcoma, we found age at diagnosis (*P* = 0.013), primary tumor volume (*P* = 0.002), presence of metastasis at diagnosis (*P* = 0.0003), histologic response to neoadjuvant chemotherapy (*P* = 0.024), and types of chemotherapy treatment, (*P* = 0.00003) were significantly associated with the OS. Other characteristics were not significantly correlated with OS.

**Fig 2:**
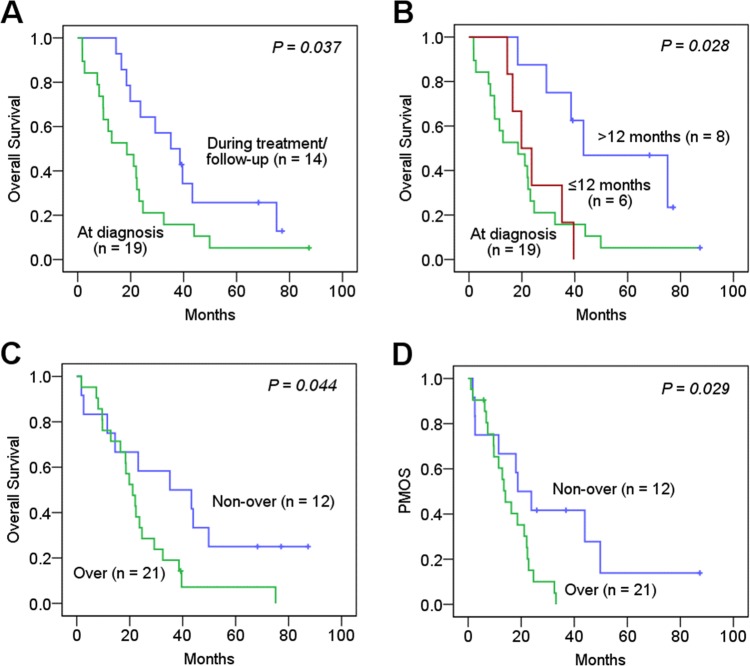
Kaplan-Meier survival curves of overall survival for patients with metastatic osteosarcoma. (A) Presence of metastasis at diagnosis or during treatment/follow-up (survival rates, 5.3% vs 21.4%, *P* = 0.037). (B) Time to metastases; at diagnosis, ≤ 12 months, > 12 months (5.3% vs 0% vs 37.5%, *P* = 0.028). (C) pFAK-Y397 overexpression and non-overexpression (4.8% vs 25%, *P* = 0.044). (D) Post metastases overall survival (PMOS) of patients with metastatic osteosarcoma stratified by pFAK-Y397 overexpression and non-overexpression (4.8% vs 25%, *P* = 0.029).

In the group of patients with metastatic osteosarcoma, survival rates, mean and median OS were significantly worse in patients with metastases at diagnosis (5.3% vs 21.4%, mean, 22 months vs 41 months; median, 19 months vs 35 months, *P* = 0.037, respectively; Fig 2A) and worse in patients having time to detection of metastases at diagnosis or within 12 months after diagnosis, than those having time to metastases more than 12 months after diagnosis (5.3% vs 0% vs 37.5%, mean, 22 months vs 25 months vs 53 months; median, 19 months vs 20 months vs 43 months, *P* = 0.028, respectively; Fig 2B). In contrast to the result of the all patient group, patients with pFAK-Y397 overexpression significantly showed worse survival rates, shorter mean and median OS than those with non-overexpression (4.8% vs 25%; mean, 24 months vs 41 months; median, 21 months vs 35 months, *P* = 0.044, respectively; Fig 2C). Other characteristics were not correlated with survival rates and OS time in this group of patients (*P* > 0.05).

We further investigated associations of characteristics and PMOS of patients with metastatic osteosarcoma. We found the patients with pFAK-Y397 overexpression significantly showed worse survival rates, and shorter mean and median PMOS than those with non-overexpressed pFAK-Y397 (4.8% vs 25%; mean PMOS, 15 months vs 32 months; median PMOS, 14 months vs 19 months, *P* = 0.029, respectively; Fig 2D). We also found that mean and median PMOS were significantly worse in patients older than 21 years of age at diagnosis (*P* = 0.011) and in patients having metastases at bone or multiple organs (lung and other organs) than at lung alone (*P* = 0.019), whereas other characteristics were not significantly correlated with OS after metastases (*P* > 0.05).

We also selectively analyzed the group of patients without metastasis at diagnosis. We found only age at diagnosis (*P* = 0.005) and neoadjuvant and adjuvant chemotherapy treatment (*P* < 0.0001) were significantly associated with the OS, but not for the other characteristics or FAK expression (S2 Table).

However, total FAK expression or total FAK/pFAK-Y397 co-expression were not significantly correlated with OS or PMOS of patients in any groups in this study. The older age group, and incomplete or absent of chemotherapy treatment were significantly associated with worse OS only in the groups of all patients and patients without metastasis at diagnosis. Larger primary tumor volume and poor histologic response were significantly associated with worse OS only in the group of all patients.

### pFAK-Y397 as an independent prognostic factor for patients with metastatic osteosarcoma

To assess the potential of using total FAK, pFAK-Y397 expression or total FAK/pFAK-Y397 co-expression in predicting the prognosis of patients with osteosarcoma and to identify significant independent prognostic factors of OS, univariate and multivariate Cox proportional hazards regression analyses were performed.

In the univariate Cox regression analyses, OS of patients in the group of all patients was affected by age at diagnosis (> 18 yrs vs ≤ 18 yrs; hazard ratio (HR) 2.60; *P* = 0.016), primary tumor volume (≥ 500 mL vs < 500 mL; HR 3.15; *P* = 0.003), presence of metastasis at diagnosis (yes vs no; HR 3.18; *P* = 0.001), histologic response to neoadjuvant chemotherapy (poor vs good; HR 3.07; *P* = 0.031) and chemotherapy treatment (either neoadjuvant or adjuvant, or no chemotherapy vs both neoadjuvant & adjuvant; HR 3.77; *P* < 0.0001), whereas gender, site of tumor, histologic subtype, total FAK, pFAK-Y397 expression and total FAK/pFAK-Y397 co-expression did not affect the survival (*P* > 0.05; S3 Table).

Interestingly, in the group of patients with metastatic osteosarcoma, OS of patients was affected by pFAK-Y397 overexpression (HR 2.32; *P* = 0.048) as well as presence of metastasis at diagnosis (HR 2.21; *P* = 0.041) and time to metastases (at diagnosis vs ≤ 12 months vs > 12 months; HR 1.77; *P* = 0.014), but not affected by the other characteristics (*P* > 0.05; S3 Table). On the other hand, only pFAK-Y397 overexpression (HR 2.60; *P* = 0.034), age at diagnosis (> 21 yrs vs ≤ 21 yrs; HR 3.32; *P* = 0.017) and site of metastases (bone or multiple organs vs lung only; HR 2.81; *P* = 0.025) affected the PMOS of patients in this group.

In the final multivariate Cox regression models for the group of all patients, only the presence of metastasis at diagnosis and chemotherapy treatment remained the most significant and independent factors for OS as shown in Table 3. The results revealed that the osteosarcoma patients who had metastasis at diagnosis or received either neoadjuvant or adjuvant, or no chemotherapy had poorer OS (HR 5.20, *P* = 0.011; HR 28.49, *P* = 0.007; respectively).

**Table 3:** Multivariate Cox regression analyses for overall survival of all patients with osteosarcoma, and for overall survival and post metastases overall survival of patients with osteosarcoma presented with metastases at diagnosis or developed metastases during treatment/follow-up.

Characteristics	All patients with osteosarcoma			Patients with metastatic osteosarcoma					
	OS			OS			PMOS		
	*P* value	HR	95% CI	*P* value	HR	95% CI	*P* value	HR	95% CI
Age at diagnosis, > 18 yrs vs ≤ 18 yrs^a^	0.988	0.0	0.0	**			0.003*	5.900	1.859–18.721
Primary tumor volume, ≥ 500 mL vs < 500 mL	0.839	1.143	0.315–4.144	**			**		
Presence of metastasis at diagnosis, yes vs no	0.011*	5.199	1.459–18.534	0.376	0.415	0.059–2.914	**		
Time to metastases, at diagnosis vs ≤ 12 months vs > 12 months	**			0.055	3.343	0.973–11.492	**		
Site of metastases, bone or multiple organs vs lung only	**			**			0.114	2.075	0.839–5.135
Histologic response, poor vs good	0.069	3.369	0.911–12.454	**			**		
Chemotherapy, either neoadjuvant or adjuvant, or no chemotherapy vs both neoadjuvant & adjuvant	0.007*	28.492	2.479–327.532	**			**		
pFAK-Y397 expression, over vs non-over	**			0.017*	3.097	1.221–7.855	0.006*	4.007	1.486–10.806

CI, confidence interval; HR, hazard ratio; OS, overall survival; PMOS, post metastases overall survival.

^a^ Mean value of age at diagnosis: patients with metastatic osteosarcoma, ≤ 21 yrs vs > 21 yrs.

* Statistically significant.

** Not available, only variables which are statistically significant in univariate Cox regression analyses were analyzed.

In the group of patients with metastatic osteosarcoma, only pFAK-Y397 expression (*P* = 0.017) remained the most significant and showed statistically independent impact on OS (Table 3). The results revealed that the risk of death for patients in this group with overexpressed pFAK-Y397 was 3.10-fold higher than those with non-overexpression (95% CI, 1.22–7.86). In addition, for PMOS, only pFAK-Y397 expression (HR 4.01; *P* = 0.006) and age at diagnosis (HR 5.90; *P* = 0.003) were independent indicators of the survival. The results revealed that patients with metastases who had overexpressed pFAK-Y397 or were older than 21 years of age at diagnosis had worse OS after metastases.

We also specifically analyzed the group of patients without metastatic osteosarcoma at diagnosis. In the univariate Cox regression analyses, only age at diagnosis (> 15 yrs vs ≤ 15 yrs; HR 3.66; *P* = 0.009) and chemotherapy treatment (either neoadjuvant or adjuvant, or no chemotherapy vs both neoadjuvant & adjuvant; HR 8.72; *P* < 0.00005) affected OS. However, in the final multivariate Cox regression analysis, only chemotherapy treatment remained the most significant and independent factor for OS (HR 6.89, *P* = 0.0004; S4 Table).

## Discussion

It has been known that metastasis is the main cause of mortality in patients with osteosarcoma and therapeutic regimens for primary osteosarcoma tumors are improbably successful in treatment of metastatic disease. Previously, several clinical characteristics have been shown to affect patient survival such as histologic response to chemotherapy [1,3,28,30–34], presence of metastasis at diagnosis [1,2,33–35], age [3,32,33,36], tumor size or volume [1,3,28,32,34], histology [29,35], and number of metastatic sites [6,37]. Nonetheless, the clinical applications of those prognostic markers in improving treatment strategies of osteosarcoma remain unclear. To our best knowledge, not many molecular markers have been evaluated for their prognostic value and none of them is being used in routine osteosarcoma treatment. There were a small number of recent studies reporting prognostic biomarkers for osteosarcoma which were associated with lung metastases and survival such as PLA2G16, MMP-2, CXCR4 and MMP9 [38–40], but none of them showed correlation with response to chemotherapy treatment. Therefore, it would be worth identifying molecular biomarkers that can be used to predict both prognosis and response to chemotherapy, especially for patients with metastatic osteosarcoma at diagnosis or during treatment/follow-up.

In the present study, we investigated the expression of FAK and its phosphorylated form, pFAK-Y397, in 53 osteosarcoma patient tissues by immunohistochemistry. No difference in staining patterns and intensities for total FAK or pFAK-Y397 was found among different histologic subtypes of osteosarcoma samples. Our results revealed that both total FAK and pFAK-Y397, were overexpressed in the majority of conventional subtypes (osteoblastic, chondroblastic and fibroblastic subtypes) and also in rare subtypes of osteosarcoma (telangiectatic and small cell subtypes). These significant findings reveal a potential for the specific targeted therapies against FAK for treatment of various subtypes of osteosarcoma [10,14,16,41]. A previous study also showed that inhibition of FAK expression by siRNA pronouncedly induced apoptosis of osteosarcoma cells in p53- and caspase-dependent manners and enhanced additive cytotoxic effect when combined with chemotherapy treatment, supporting FAK function as a potent inhibitor of apoptosis as well as an attractive therapeutic target in osteosarcoma [41]. The total FAK was expressed in both the nuclei and cytoplasm of osteosarcoma, but pFAK-Y397 was expressed mainly in cytoplasm. The cytoplasmic expression pattern of pFAK-Y397 was consistent with previous reports in osteosarcoma [26,27]. In addition to our results, published studies have reported nuclear expression of pFAK-Y397 in other types of cancers, such as colorectal, esophageal, pancreatic and breast cancers [13,42]. It is likely that context-dependent environments among different cell types may contribute to distinct subcellular localizations of phosphorylated FAK.

There were a small number of published literatures concerning FAK expression and clinical outcomes of osteosarcoma [26,27]. Moreover, all previous studies only focused on patients without metastatic disease at diagnosis, and excluded those with metastases at diagnosis. One of the studies showed only the correlation of FAK with response to chemotherapy, but the authors failed to show a correlation between the combined biomarkers (including FAK) and OS of patients [26]. Another recent study specifically investigated FAK expression in osteoblastic-subtype osteosarcoma of patients and found the correlation of co-expressed FAK and pFAK with survival, but not the response to chemotherapy of patients [27]. As a number of patients with osteosarcoma may present with metastatic tumor at diagnosis. None of these findings may be applicable to treatment strategies for this group of patients, because of lack of evidence-based correlation studies.

Therefore, in our study, we aimed to compare total FAK and pFAK-Y397 expression with clinicopathologic characteristics and OS of patients with various histologic subtypes of osteosarcoma who presented with metastatic disease at diagnosis or developed metastases during treatment/follow-up, as well as in all patients group including patients without metastasis at diagnosis. Remarkably, we found overexpression of pFAK-Y397 in primary tumor tissue was statistically correlated with poor histologic response to neoadjuvant chemotherapy in all patients with osteosarcoma, regardless of the presence of metastasis at diagnosis or during treatment/follow-up, or without metastasis at diagnosis. Our study result was consistent with the previous report of association between pFAK-Y397 expression profile and poor response to chemotherapy of osteosarcoma patients without metastatic disease at diagnosis [26]. Furthermore, our result has shown that pFAK-Y397 expression might be used to predict histologic response to chemotherapy treatment for all osteosarcoma patients of all stages of disease, and histologic subtypes. It was previously shown that patients with a poor response to neoadjuvant chemotherapy were at higher risk for developing metastases [43]. Our data also suggest that patients with overexpressed pFAK-Y397 should be considered for treatment with tailor-made modified chemotherapy regimens or novel targeted therapy against FAK, for increasing chance of better histologic response and outcomes.

More interestingly, in our Kaplan-Meier and multivariate regression analyses, we found pFAK-Y397 overexpression, but not total FAK expression or combined total FAK/pFAK-Y397 co-expression, was significantly associated with worse OS and worse PMOS in osteosarcoma patients with metastases at diagnosis or developed metastases during treatment/follow-up. To our knowledge, this is the first report of such a correlation in literatures. However, we did not find the same correlation in patients without metastasis at diagnosis in our cohort study, which contrasted with the result of the previous study of combined FAK and pFAK co-expression profile and OS of osteoblastic osteosarcoma patients [27]. The reason for this discrepancy was unknown. However, it should be noted that the previous study only recruited osteoblastic-subtype osteosarcoma cases, whereas we included various histologic subtypes of osteosarcoma cases in which nearly half of the cases were non-osteoblastic subtype cases. Histologic subtype has been shown to be correlated with histologic response to chemotherapy and with poor prognosis of osteosarcoma [29]. A recent study also reported that patients with metastatic osteosarcoma, who had chondroblastic subtype, was correlated with worse OS than those with other histologic subtypes, including osteoblastic subtype [35]. Our results suggest that the expression of pFAK-Y397 alone may be an independent predictive biomarker for poor OS of osteosarcoma patients who develop metastases.

In accordance with our findings, it was also demonstrated in the recent study of advanced gastric cancer suggesting that pFAK-Y397, but not total FAK expression, may be able to predict recurrence of gastric carcinoma [25]. Similarly, pFAK-Y397 overexpression, but not total FAK, had an impact on OS and positively correlated with lymph node and distant metastases in advanced stage serous epithelial ovarian cancer [23]. Therefore, total FAK expression does not necessarily influence prognosis of these cancers including metastatic osteosarcoma, whereas pFAK-Y397 does.

Moreover, we found pFAK-Y397 overexpression was independently associated with shorter PMOS in patients with metastatic osteosarcoma. Having time to develop metastases less than 12 months after diagnosis was shown to be significantly associated with poorer OS of patients in this group [44]. These results implicate that patients who develop metastases within the first year of diagnosis or have pFAK-Y397 overexpression might have more aggressive disease outcome. The role of FAK in cell migration is well-characterized, and tumor progression and metastasis can be promoted by FAK signaling pathways through their regulation of cell migration, invasion, epithelial to mesenchymal transition and angiogenesis [9,10]. There was evidence from a previous study that increased phosphorylated form of FAK and Src via activation by the extracellular matrix glycoprotein, Spondin 1, promoted cell invasiveness and pulmonary metastatic progression of osteosarcoma [45]. Thus, overexpression of pFAK-Y397 may participate in the worse progression of osteosarcoma patients with metastases.

## Conclusions

We have shown that total FAK and pFAK-Y397 proteins were commonly overexpressed in various subtypes of osteosarcoma. The results of our study revealed that certain clinicopathologic factors such as presence of metastasis at diagnosis, chemotherapy treatment and age at diagnosis may be independent indicators for OS of patients with osteosarcoma depending on each specific group. Most importantly, pFAK-Y397 overexpression alone, but not total FAK expression or total FAK/pFAK-Y397 co-expression, can be an independent biomarker predicting OS and PMOS for patients who have metastases at diagnosis or during treatment/follow-up. Moreover, pFAK-Y397 may serve as a routine clinical biomarker of response to chemotherapy for patients with various histologic subtypes of osteosarcoma, regardless of the presence of metastasis or not. Our data suggest that pFAK-Y397 would be an attractively potential therapeutic target for personalized treatment of patients with metastatic osteosarcoma, as well as for further development of novel targeted therapy by clinical trials.

## Supporting information

S1 Table Relationships between clinicopathologic characteristics and total FAK, pFAK-Y397 expression in primary osteosarcoma tissues of patients without metastatic osteosarcoma at diagnosis. (PDF)

S2 Table Kaplan-Meier survival analyses for overall survival of patients without metastatic osteosarcoma at diagnosis. (PDF)

S3 Table Univariate Cox regression analyses for overall survival of all patients with osteosarcoma, and for overall survival and post metastases overall survival of patients with osteosarcoma presented with metastases at diagnosis or developed metastases during treatment/follow-up. (PDF)

S4 Table Univariate and multivariate Cox regression analyses for overall survival of patients without metastatic osteosarcoma at diagnosis. (PDF)
