# Correction: pFAK-Y397 overexpression as both a prognostic and a predictive biomarker for patients with metastatic osteosarcoma

**DOI:** 10.1371/journal.pone.0188991

**Published:** 2017-11-29

**Authors:** The PLOS ONE Staff

There is an error in Table 2. Two cells in the row corresponding to patients under 18 years of age at diagnosis were incorrectly shifted to the left. The value “6” should appear in the “*n*” column and “16 (3.1–28.4)” should be listed in the “Mean months (95% CI)” column. The publisher apologizes for this error. Please see the corrected Table 2 here.

**Table 2:** Kaplan-Meier survival analyses for overall survival of all patients with osteosarcoma and for overall survival and post metastases overall survival of patients with osteosarcoma presented with metastases at diagnosis or developed metastases during treatment/follow-up.

Characteristics	All patients with osteosarcoma (*n* = 53)			Patients with metastatic osteosarcoma (*n* = 33)					
	OS			OS			PMOS		
	*n*	Mean months (95% CI)	*P* value^d^	*n*	Mean months (95% CI)	*P* value^d^	*n*	Mean months (95% CI)	*P* value^d^
Age at diagnosis^a^									
≤ 18 yrs	42	56 (40.9–70.9)	0.013*	27	34 (23.9–43.3)	0.062	27	24 (15.8–32.4)	0.011*
> 18 yrs	11	19 (8.4–28.9)		6	16 (3.1–28.4)		6	9 (3.3–15.2)	
Gender									
Male	36	51 (34.7–66.8)	0.780	27	32 (21.6–42.0)	0.485	27	23 (14.0–31.4)	0.389
Female	17	45 (24.2–66.2)		6	24 (16.1–31.8)		6	16 (8.9–23.5)	
Site of tumor									
Femur	31	58 (39.2–77.2)	0.326	16	32 (20.5–43.7)	0.661	16	26 (14.7–37.6)	0.208
Tibia or fibula or others	22	40 (24.2–55.0)		17	28 (16.5–39.6)		17	16 (9.0–23.6)	
Histologic subtype									
Non osteoblastic	25	62 (41.1–81.9)	0.102	16	36 (24.0–47.9)	0.140	16	22 (15.3–28.7)	0.451
Osteoblastic	28	37 (22.6–51.7)		17	24 (13.8–33.4)		17	19 (8.9–29.0)	
Primary tumor volume^b^									
< 500 mL	26	69 (49.2–89.3)	0.002*	12	43 (26.4–59.6)	0.074	12	28 (12.6–43.8)	0.374
≥ 500 mL	19	24 (15.2–33.0)		14	24 (14.4–32.6)		14	20 (11.9–27.1)	
Presence of metastasis at diagnosis									
No	34	65 (47.1–82.8)	0.0003*	14	41 (28.5–52.8)	0.037*	14	18 (12.3–24.4)	0.882
Yes	19	22 (12.5–30.7)		19	22 (12.5–30.7)		19	21 (12.1–30.4)	
Time to metastases									
> 12 months				8	53 (37.1–69.4)	0.028*	8	21 (11.9–29.4)	0.688
≤ 12 months				6	25 (16.6–33.1)		6	16 (7.1–25.0)	
At diagnosis				19	22 (12.5–30.7)		19	21 (12.1–30.4)	
Site of metastases									
Lung only				25	34 (24.0–44.7)	0.050	25	25 (15.6–33.6)	0.019*
Bone or multiple organs				8	18 (8.5–26.5)		8	12 (6.7–16.4)	
Histologic response^c^									
Good	13	87 (59.2–114.7)	0.024*	5	37 (17.0–57.5)	0.379	5	25 (7.5–42.3)	0.419
Poor	22	41 (27.4–53.8)		16	29 (19.6–38.8)		16	20 (14.1–25.2)	
Chemotherapy									
Both neoadjuvant & adjuvant	34	65 (48.0–81.8)	0.00003*	21	34 (25.0–43.8)	0.133	21	23 (16.6–29.0)	0.183
Either neoadjuvant or adjuvant, or no chemotherapy	19	20 (9.4–30.0)		12	22 (8.3–35.1)		12	17 (3.8–29.9)	
Total FAK expression									
Non-over	5	66 (21.1–109.9)	0.449	2	29 (17.5–40.9)	0.958	2	21 (15.2–26.6)	0.962
Over	48	45 (32.6–57.0)		31	30 (21.3–39.4)		31	21 (13.8–29.0)	
pFAK-Y397 expression									
Non-over	20	60 (37.5–82.5)	0.258	12	41 (23.2–58.1)	0.044*	12	32 (14.9–48.6)	0.029*
Over	33	40 (26.2–54.6)		21	24 (16.4–32.1)		21	15 (11.5–19.4)	
Total FAK/pFAK-Y397 co-expression									
Non-over/non-over	5	66 (21.1–109.9)	0.502	2	29 (17.5–40.9)	0.098	2	21 (15.2–26.6)	0.071
Over/non-over	15	49 (29.0–68.3)		10	43 (22.3–63.6)		10	34 (14.0–53.8)	
Over/over	33	40 (26.2–54.6)		21	24 (16.4–32.1)		21	15 (11.5–19.4)	

CI, confidence interval; OS, overall survival; PMOS, post metastases overall survival.

^a^ Mean value of age at diagnosis: patients with metastatic osteosarcoma, ≤ 21 yrs vs > 21 yrs.

^b^ All patients with osteosarcoma, evaluated only in 45 patients (8 patients, no preoperative MRI); patients with metastatic osteosarcoma, evaluated only in 26 patients (7 patients, no preoperative MRI).

^c^ All patients with osteosarcoma, evaluated only in 35 patients who received neoadjuvant chemotherapy (5 patients, no neoadjuvant chemotherapy; 5 patients, no chemotherapy; 8 patients, no data); patients with metastases, evaluated only in 21 patients who received neoadjuvant chemotherapy (5 patients, no neoadjuvant chemotherapy; 5 patients, no chemotherapy; 2 patients, no data).

^d^ Log rank (Mantel-Cox) test.

* Statistically significant.

## References

[1] Thanapprapasr K, Nartthanarung A, Thanapprapasr D, Jinawath A (2017) pFAK-Y397 overexpression as both a prognostic and a predictive biomarker for patients with metastatic osteosarcoma. PLoS ONE 12(8): e0182989 https://doi.org/10.1371/journal.pone.0182989 28846700 10.1371/journal.pone.0182989 PMC5573209

