# Supplementary material for: pFAK-Y397 overexpression as both a prognostic and a predictive biomarker for patients with metastatic osteosarcoma
Source: PLoS One. 2017 Aug 28;12(8):e0182989. doi: 10.1371/journal.pone.0182989 (PMC5573209; doi:10.1371/journal.pone.0182989)
Supplement: S1 Table — (PDF) [file pone.0182989.s001.pdf]

S1 Table. Relationships between clinicopathologic characteristics and total FAK, pFAK-Y397 expression in primary osteosarcoma tissues of patients without metastatic osteosarcoma at diagnosis. (non-overexpression = negative or weak or moderate expression)

**Characteristics**	**Total FAK expression**			**pFAK-Y397 expression**		
	**Non-over (*n* = 4) *n* (%)**	**Over (*n* = 30) *n* (%)**	***P* value[c]**	**Non-over (*n* = 13) *n* (%)**	**Over (*n* = 21) *n* (%)**	***P* value[c]**
Age at diagnosis						
≤ 15 yrs	3 (8.8)	18 (52.9)	1.000	10 (29.4)	11 (32.4)	0.276
> 15 yrs	1 (2.9)	12 (35.3)		3 (8.8)	10 (29.4)	
Gender						
Male	4 (11.8)	15 (44.1)	0.113	9 (26.5)	10 (29.4)	0.217
Female	0 (0.0)	15 (44.1)		4 (11.8)	11 (32.4)	
Site of tumor						
Femur	3 (8.8)	18 (52.9)	1.000	7 (20.6)	14 (41.2)	0.491
Tibia or fibula or others	1 (2.9)	12 (35.3)		6 (17.6)	7 (20.6)	
Histologic subtype						
Non osteoblastic	2 (5.9)	15 (44.1)	1.000	7 (20.6)	10 (29.4)	0.724
Osteoblastic	2 (5.9)	15 (44.1)		6 (17.6)	11 (32.4)	
Primary tumor volume[a]						
< 500 mL	2 (6.9)	19 (65.5)	1.000	8 (27.6)	13 (44.8)	1.000
≥ 500 mL	1 (3.4)	7 (24.1)		3 (10.3)	5 (17.2)	
Histologic response[b]						
Good	4 (17.4)	7 (30.4)	0.037*	7 (30.4)	4 (17.4)	0.009*
Poor	0 (0.0)	12 (52.2)		1 (4.3)	11 (47.8)	

[a] Evaluated only in 29 patients (5 patients, no preoperative MRI).

[b] Evaluated only in 23 patients who received neoadjuvant chemotherapy (3 patients, no neoadjuvant chemotherapy; 1 patients, no chemotherapy; 7 patients, no data).

[c] *P* value determined using the Pearson Chi-square test or Fisher's exact test.

* Statistically significant.
