# Supplementary material for: pFAK-Y397 overexpression as both a prognostic and a predictive biomarker for patients with metastatic osteosarcoma
Source: PLoS One. 2017 Aug 28;12(8):e0182989. doi: 10.1371/journal.pone.0182989 (PMC5573209; doi:10.1371/journal.pone.0182989)
Supplement: S2 Table — (PDF) [file pone.0182989.s002.pdf]

S2 Table. Kaplan-Meier survival analyses for overall survival of patients without metastatic osteosarcoma at diagnosis.

Characteristics	*n*	Mean months (95% CI)	*P* value[c]
Age at diagnosis			
≤ 15 yrs	21	82 (60.4 – 104.1)	0.005*
> 15 yrs	13	28 (18.1 – 38.4)	
Gender			
Male	19	75 (51.9 – 98.4)	0.135
Female	15	49 (25.7 – 72.1)	
Site of tumor			
Femur	21	72 (47.9 – 96.4)	0.722
Tibia or fibula or others	13	56 (34.8 – 77.2)	
Histologic subtype			
Non osteoblastic	17	82 (57.2 – 106.8)	0.052
Osteoblastic	17	44 (24.5 – 64.1)	
Primary tumor volume[a]			
< 500 mL	21	75 (52.4 – 97.4)	0.138
≥ 500 mL	8	32 (13.9 – 50.5)	
Histologic response[b]			
Good	11	99 (71.1 – 126.2)	0.127
Poor	12	58 (37.3 – 77.9)	
Chemotherapy			
Both neoadjuvant & adjuvant	23	86 (65.7 – 106.1)	<0.0001*
Either neoadjuvant or adjuvant, or no chemotherapy	11	19 (10.5 – 28.1)	
Total FAK expression			
Non-over	4	76 (25.6 – 126.6)	0.634
Over	30	58 (41.7 – 74.7)	
pFAK-Y397 expression			
Non-over	13	72 (44.0 – 100.7)	0.557
Over	21	54 (34.6 – 74.1)	
Total FAK/pFAK-Y397 co-expression			
Non-over/non-over	4	76 (25.6 – 126.6)	0.820
Over/non-over	9	58 (32.6 – 83.4)	
Over/over	21	54 (34.6 – 74.1)	

CI, confidence interval.

[a] Evaluated only in 29 patients (5 patients, no preoperative MRI).

[b] Evaluated only in 23 patients who received neoadjuvant chemotherapy (3 patients, no neoadjuvant chemotherapy; 1 patients, no chemotherapy; 7 patients, no data).

[c] Log rank (Mantel-Cox) test.

* Statistically significant.
