# Supplementary material for: pFAK-Y397 overexpression as both a prognostic and a predictive biomarker for patients with metastatic osteosarcoma
Source: PLoS One. 2017 Aug 28;12(8):e0182989. doi: 10.1371/journal.pone.0182989 (PMC5573209; doi:10.1371/journal.pone.0182989)
Supplement: S3 Table — (PDF) [file pone.0182989.s003.pdf]

S3 Table. Univariate Cox regression analyses for overall survival of all patients with osteosarcoma, and for overall survival and post metastases overall survival of patients with osteosarcoma presented with metastases at diagnosis or developed metastases during treatment/follow-up.

Characteristics	All patients with osteosarcoma (*n* = 53)			Patients with metastatic osteosarcoma (*n* = 33)					
	OS			OS			PMOS		
	P value	HR	95% CI	*P* value	HR	95% CI	*P* value	HR	95% CI
Age at diagnosis, > 18 yrs vs ≤ 18 yrs[a]	0.016*	2.595	1.192 – 5.648	0.070	2.341	0.934 – 5.869	0.017*	3.319	1.244 – 8.855
Gender, female vs male	0.780	1.103	0.554 – 2.197	0.486	1.389	0.551 – 3.504	0.393	1.493	0.595 – 3.747
Site of tumor, tibia or fibula or others vs femur	0.328	1.382	0.723 – 2.640	0.662	1.178	0.565 – 2.457	0.212	1.598	0.765 – 3.338
Histologic subtype, osteoblastic vs non osteoblastic	0.106	1.720	0.891 – 3.323	0.145	1.734	0.828 – 3.631	0.453	1.332	0.630 – 2.813
Primary tumor volume, ≥ 500 mL vs < 500 mL	0.003*	3.150	1.471 – 6.744	0.081	2.216	0.906 – 5.422	0.378	1.476	0.622 – 3.503
Presence of metastasis at diagnosis, yes vs no	0.001*	3.181	1.641 – 6.167	0.041*	2.207	1.032 – 4.719	0.882	1.060	0.491 – 2.288
Time to metastases, at diagnosis vs ≤ 12 months vs > 12 months				0.014*	1.769	1.124 – 2.786	0.646	1.115	0.701 – 1.772
Site of metastases, bone or multiple organs vs lung only				0.057	2.303	0.977 – 5.430	0.025*	2.811	1.136 – 6.951
Histologic response, poor vs good	0.031*	3.070	1.110 – 8.489	0.384	1.646	0.536 – 5.052	0.425	1.585	0.512 – 4.913
Chemotherapy, either neoadjuvant or adjuvant, or no chemotherapy vs both neoadjuvant & adjuvant	<0.0001*	3.774	1.934 – 7.364	0.138	1.774	0.832 – 3.782	0.188	1.669	0.778 – 3.582
Total FAK expression, over vs non-over	0.453	1.574	0.482 – 5.140	0.958	0.962	0.225 – 4.115	0.962	1.036	0.243 – 4.419
pFAK-Y397 expression, over vs non-over	0.261	1.486	0.745 – 2.963	0.048*	2.323	1.006 – 5.362	0.034*	2.601	1.072 – 6.307
Total FAK/pFAK-Y397 co-expression, over/over vs over/non-over vs non-over/non-over	0.250	1.355	0.807 – 2.274	0.120	1.751	0.864 – 3.551	0.091	1.879	0.904 – 3.903

CI, confidence interval; HR, hazard ratio; OS, overall survival; PMOS, post metastases overall survival.

[a] Patients with metastases, > 21 yrs vs ≤ 21 yrs (the mean value of age at diagnosis).

* Statistically significant.
