# Supplementary material for: pFAK-Y397 overexpression as both a prognostic and a predictive biomarker for patients with metastatic osteosarcoma
Source: PLoS One. 2017 Aug 28;12(8):e0182989. doi: 10.1371/journal.pone.0182989 (PMC5573209; doi:10.1371/journal.pone.0182989)
Supplement: S4 Table — (PDF) [file pone.0182989.s004.pdf]

S4 Table. Univariate and multivariate Cox regression analyses for overall survival of patients without metastatic osteosarcoma at diagnosis.

Characteristics	*P* value	HR	95% CI
Univariate			
Age at diagnosis, > 15 yrs vs ≤ 15 yrs	0.009*	3.659	1.390 – 9.633
Gender, female vs male	0.143	1.965	0.796 – 4.851
Site of tumor, tibia or fibula or others vs femur	0.722	1.179	0.476 – 2.920
Histologic subtype, osteoblastic vs non osteoblastic	0.059	2.468	0.965 – 6.314
Primary tumor volume, ≥ 500 mL vs < 500 mL	0.149	2.277	0.745 – 6.962
Histologic response, poor vs good	0.143	2.798	0.707 – 11.073
Chemotherapy, either neoadjuvant or adjuvant, or no chemotherapy vs both neoadjuvant & adjuvant	<0.00005*	8.719	3.073 – 24.732
Total FAK expression, over vs non-over	0.636	1.427	0.327 – 6.227
pFAK-Y397 expression, over vs non-over	0.558	1.328	0.514 – 3.434
Total FAK/pFAK-Y397 co-expression, over/over vs over/non-over vs non-over/non-over	0.530	1.241	0.632 – 2.435
Multivariate			
Age at diagnosis, > 15 yrs vs ≤ 15 yrs	0.086	2.410	0.884 – 6.571
Chemotherapy, either neoadjuvant or adjuvant, or no chemotherapy vs both neoadjuvant & adjuvant	0.0004*	6.893	2.360 – 20.136

CI, confidence interval; HR, hazard ratio.
* Statistically significant.
